# Influence of climate variability and seasonal trends on malaria incidence in Dar es Salaam, Tanzania using generalized additive models

**DOI:** 10.1017/S095026882610185X

**Published:** 2026-06-23

**Authors:** Iddi Mapande, January G. Msemakweli, Katherine C. Lan, Victor Okpanachi, Hussein Mohamed, Rajendra P. Shrestha

**Affiliations:** 1 Department of Environmental and Occupational Health, School of Public Health and Science Muhimbili University of Health and Allied Sciences, Dar es Salaam, Tanzania; 2 Department of Epidemiology, Johns Hopkins Bloomberg School of Public Health, Baltimore, MD, USA; 3 Department of Environmental Health Science, The University of Arizona Mel and Enid Zuckerman College of Public Health, Tucson, AZ, USA; 4 Department of Food, Agriculture and Natural Resources, Development and Sustainability, Asian Institute of Technology School of Environment Resources and Development, Thailand

**Keywords:** climate variability, Dar es Salaam, ecological time series, generalized additive model, malaria incidence

## Abstract

While malaria transmission in coastal East Africa is strongly shaped by climatic variability, few studies examine long-term interactions in rapidly urbanizing settings. This study evaluated the impact of climate and seasonal trends on malaria incidence in Dar es Salaam, Tanzania (2014–2024). Monthly cases and meteorological data were analyzed using seasonal-trend decomposition (STL) and generalized additive models (GAMs) to quantify nonlinear and lagged climatic associations. Over the decade, malaria incidence declined sharply from >130 cases per 10,000 in 2014 to <30 by 2023. However, strong seasonal peaks persisted, with STL revealing consistent annual surges during April–June following the rainy season. GAM analysis identified rainfall as the dominant climatic driver, demonstrating significant 1- and 2-month lagged effects (p < 0.001). Daytime (1-month lag) and night-time (2-month lag) temperatures showed non-linear associations, peaking in incidence at optimal mosquito-development temperatures (~30–31°C). Despite substantial incidence declines, transmission remains highly climate-sensitive. Driven primarily by lagged rainfall and temperature effects rather than current-month conditions, these dynamics underscore the urgent need for climate-informed early warning systems and targeted seasonal interventions in coastal urban environments.

## Introduction

Malaria remains a critical global public health challenge, with shifting environmental and climatic conditions fundamentally altering its transmission dynamics. Globally, approximately 263 million cases and nearly 600000 deaths were reported in recent years, with transmission risks uniquely sensitive to anthropogenic climate change [1, 2]. The burden is profoundly unequal; sub-Saharan Africa (SSA) bears approximately 95% of all malaria cases. Recent ecological models project that warming temperatures, shifting rainfall patterns, and the increasing frequency of extreme, disruptive weather events will significantly expand *Anopheles* vector habitats across the continent, potentially driving tens of millions of new cases while simultaneously interrupting traditional vector control programmes [3, 4]. Within this highly vulnerable regional context, Tanzania remains one of the most severely affected nations. Contributing to over 4% of global malaria mortality, Tanzania faces an escalating threat as changing microclimatic conditions alter localized transmission epidemiology and challenge the long-term efficacy of standard interventions, making climate-informed surveillance in its rapidly urbanizing coastal areas urgently necessary [5, 6].

The transmission of these cases is intricately linked to meteorological factors such as temperature, rainfall, and relative humidity, which dictate the duration of the mosquito and parasite life cycles [7]. The Intergovernmental Panel on Climate Change (IPCC) reports with high confidence that climate variability has already driven malaria epidemics across East Africa, altering the historical range of the *Anopheles* mosquito and putting tens of millions more individuals at risk [8, 9]. In Tanzania, where *Plasmodium falciparum* accounts for 96% of cases [10], these shifting epidemiological patterns are further complicated by extreme weather events, such as unprecedented flooding and drought [11, 12]. These climate-driven disruptions threaten to reverse recent gains in vector control and escalate transmission risks, particularly among vulnerable populations such as children under five and pregnant women [13].

Despite these escalating risks, the specific mechanisms by which shifting climate patterns and delayed weather effects influence malaria transmission in Dar es Salaam remain under-researched. The persistence of climate-sensitive disease transmission, even amidst a massive overall decline in urban malaria cases, makes understanding these non-linear interactions essential for future public health planning. Therefore, this study aims to evaluate temporal trends in malaria incidence in Dar es Salaam over a 10-year period (2014–2024) and to use generalized additive models (GAMs) to assess the non-linear, lagged effects of critical climatic factors on transmission dynamics.

## Materials and methods

### Study design

This ecological time series study analysed the temporal patterns and trends of malaria incidence. It investigated the relationship between climatic factors and malaria incidence in Dar es Salaam, Tanzania, from January 2014 to October 2024.

### Study area

**Figure 1.**
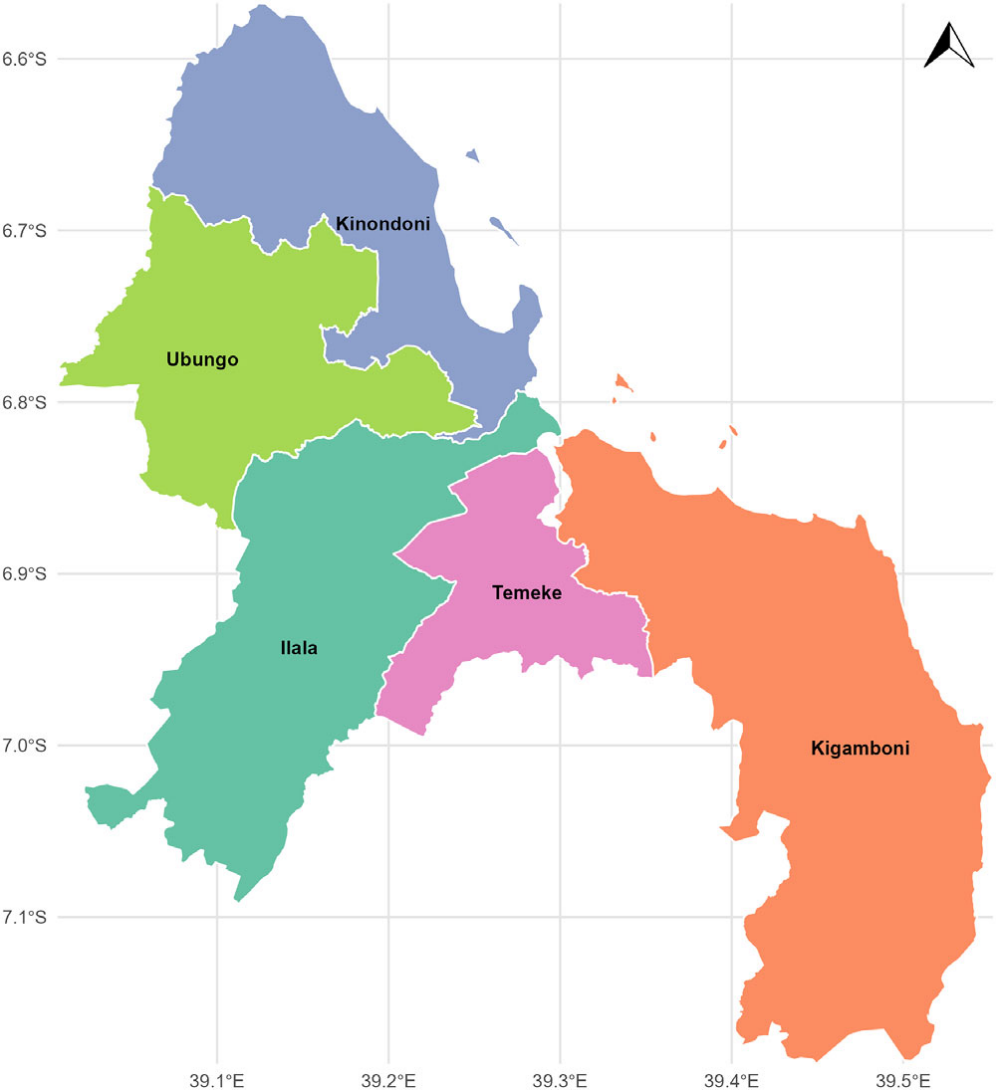
Map of study area (Authors’ own contribution). Figure 1. long description.

Dar es Salaam is one of the 31 regions on the mainland of Tanzania, divided into five districts and 90 wards, including Ilala, Kinondoni, Temeke, Ubungo, and Kigamboni (Figure 1). It is located along the southwest coast of the Indian Ocean on a flat coastal plain, and covers an area of approximately 1350 km^2^. It experiences warm, humid weather influenced by the monsoon season. The northeast monsoon affects the city between December and March, whereas the southeast monsoon occurs from May to October. Due to its proximity to the equator, the yearly mean air temperature is approximately 26°C, with modest seasonal fluctuations. The coolest months are May through September, when the average air temperature is around 24°C, while the warmest months are December through March, when the average air temperature is around 28°C [14].

### Data sources

#### Malaria data

Confirmed monthly malaria cases for each district over the past 10 years (January 2014–October 2024) were collected from the District Health Information System (DHIS2). The data were obtained from outpatient, inpatient, and reproductive and child health services. Prior to analysis, rigorous quantitative data quality checks were performed to ensure temporal completeness and internal consistency. Time series plots were visually inspected for anomalous spikes or reporting errors, and the dataset was complete with no extreme outliers requiring exclusion. This aggregate data included confirmed malaria cases categorized into groups of children under 5 years of age, patients aged above 5 years, and pregnant women.

#### Climate data

Climate data, including monthly daytime and night-time temperatures, rainfall, and relative humidity, from January 2014 to October 2024, were collected from the Julius Nyerere International Airport station operated by the Tanzania Meteorological Authority (TMA), Dar es Salaam Region Division.

#### Population data

The annual population data for Dar es Salaam and its districts, spanning 2014–2024, were obtained from the National Bureau of Statistics (NBS).

Due to the aggregated and secondary nature of the health and meteorological records, the raw data were acquired upon formal request from the respective authorities. To ensure transparency and reproducibility, the finalized, compiled dataset utilized for all modelling in this study has been made publicly accessible as detailed in the Data Availability Statement.

### Data analysis

#### Temporal patterns and trends analysis

Monthly malaria incidence (I) per 10000 person-months was calculated by dividing the total confirmed malaria cases (A) by the total population at risk (N) and multiplying the result by 10000:

$$I=\left(A/N\right)\times 10,000\;\text{person}-\text{months}$$

The mean and standard deviation of monthly malaria incidence per 10000 person-months were derived for each calendar year.

#### Seasonal trend decomposition

A seasonal-trend decomposition using locally weighted (STL) regression was applied to the time series of malaria incidence to identify seasonal relationships, interannual patterns, and residual variability. The structure of the STL model was as follows:

$$Y_{t}=S_{t}+T_{t}+R_{t}$$

where *Y*_t_ represents local malaria incidence with logarithmic transformation; *S*_t_ represents the seasonal component estimated using a locally weighted regression (LOESS) method to capture the periodic fluctuations; *T*_t_ represents the trend component estimated using another LOESS smoothing to capture the long-term direction; *R*_t_ represents the residual component calculated as the difference between the observed time series and the sum of the estimated trend and seasonal components; and *t* denotes a time index in months.

#### Climate–malaria relationship analysis

Generalized additive models (GAMs) were selected for this study to examine the relationship between climate variables and malaria incidence due to their ability to account for seasonal patterns, non-linear trends, and delayed environmental exposures. GAMs and distributed lag models are highly robust methods increasingly utilized in modern spatial epidemiology to quantify the complex, delayed physiological effects of climate on vector-borne disease transmission [15, 16]. GAMs can capture threshold or declining effects more accurately than a straightforward linear regression by modelling each climatic predictor with its smooth function. Using penalized splines to prevent overfitting keeps the model adaptable and comprehensible for any climatic element. To capture potential delayed climatic effects, a systematic lag analysis was conducted testing extended lag windows of up to 4 months (approximately 16 weeks). However, Akaike Information Criterion (AIC) comparisons confirmed that incorporating 4-month lags did not improve model performance (AIC increased from 851.48 to 852.31), and none of the 4-month climatic variables were statistically significant. Consequently, to maintain the most parsimonious fit, the final model was restricted to lags ranging from 0 to 3 months. Because the dependent variable was transformed into a continuous incidence rate, the GAM was fitted using a Gaussian distribution with an identity link function, which precludes the need for a count-based distribution.

To prevent overfitting and allow the model to automatically shrink non-informative predictors toward zero, all smooth terms were fitted using shrinkage cubic regression splines (bs = ‘cs’), which permit effective degrees of freedom (edf) to collapse when variables have weak evidence.

The GAM model formula was as follows:

$$I_{t}=\beta_{0}+\sum_{i=1}^{4}\sum_{l=0}^{3}s_{i,l}\left(X_{i,t-l},k=10\right)+s_{\text{time}}\left(\mathrm{Tim}e_{t},k=12\right)+\epsilon_{t}$$

where $I_{t}$ is the continuous malaria incidence per 10000 population at month *t*; $\beta_{0}$ is the model intercept; $s_{i,l}$ represents the shrinkage smooth functions for the four climatic predictors (daytime temperature, night-time temperature, monthly rainfall, and relative humidity) assessed at lags 0, 1, 2, 3, and 4 months with a basis dimension k of 10; $s_{\text{time}}\;$ is the penalized temporal spline (year-month) with *k* = 12; and $\epsilon_{t}$ is the Gaussian error term.

Model diagnostics were rigorously evaluated using the mgcv package in R. The gam. Check() function was used to assess the normality of residuals and homoscedasticity, and to verify that the chosen basis dimensions were sufficiently large to capture the non-linearity without overfitting. Furthermore, autocorrelation function (ACF) and partial autocorrelation function (PACF) plots of the model residuals were examined to ensure the temporal spline adequately controlled for residual temporal autocorrelation. Additionally, potential collinearity among the non-linear terms was evaluated using the concurvity() function, ensuring the smooth terms did not excessively concur.

To include unmeasured, long-term confounding factors such as insecticide-treated nets (ITNs), indoor residual spraying (IRS), and urbanization, a temporal trend term was included as a penalized spline in the model. Because granular, monthly data on vector control coverage were unavailable at the district level for the decade, this highly flexible temporal spline was utilized as a statistical proxy. By absorbing the gradual, non-linear, and long-term declines in malaria incidence driven by these interventions, the model effectively partials out this variance. This ensures that the remaining fluctuations explained by the climatic predictors represent true short-term, seasonal climate sensitivities rather than spurious correlations with long-term public health successes.

#### Statistical software

All analyses were conducted using R software, version 4.5.1 (© R Foundation for Statistical Computing). The mgcv package was used for GAM analysis, and custom scripts were developed for STL decomposition and preliminary data visualization.

## Results

### Monthly and annual trends of malaria incidence

**Figure 2.**
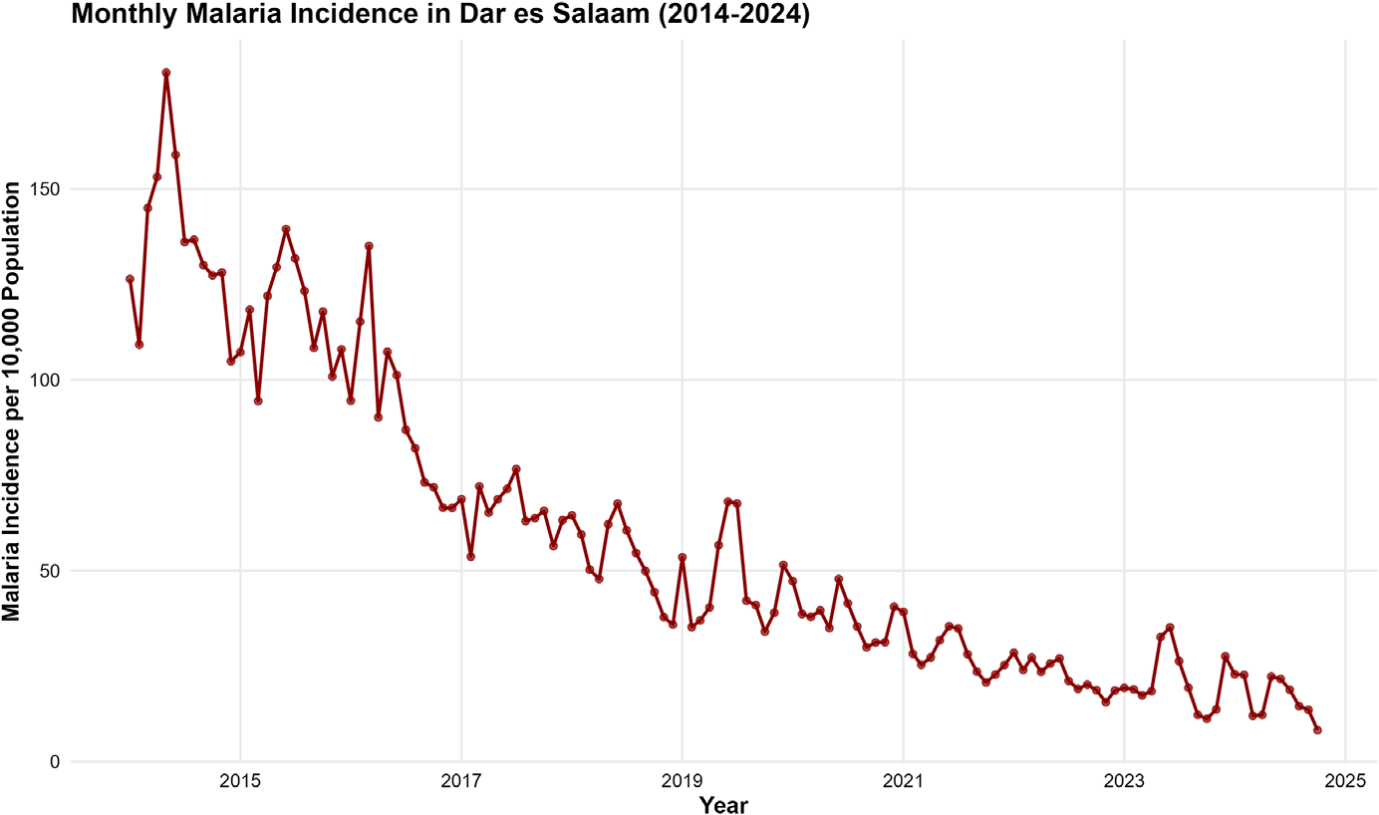
Monthly trend of malaria incidence in Dar es Salaam (2014–2024). The overall downward trend is highly statistically significant (*p* < 0.001). Figure 2. long description.

**Figure 3.**
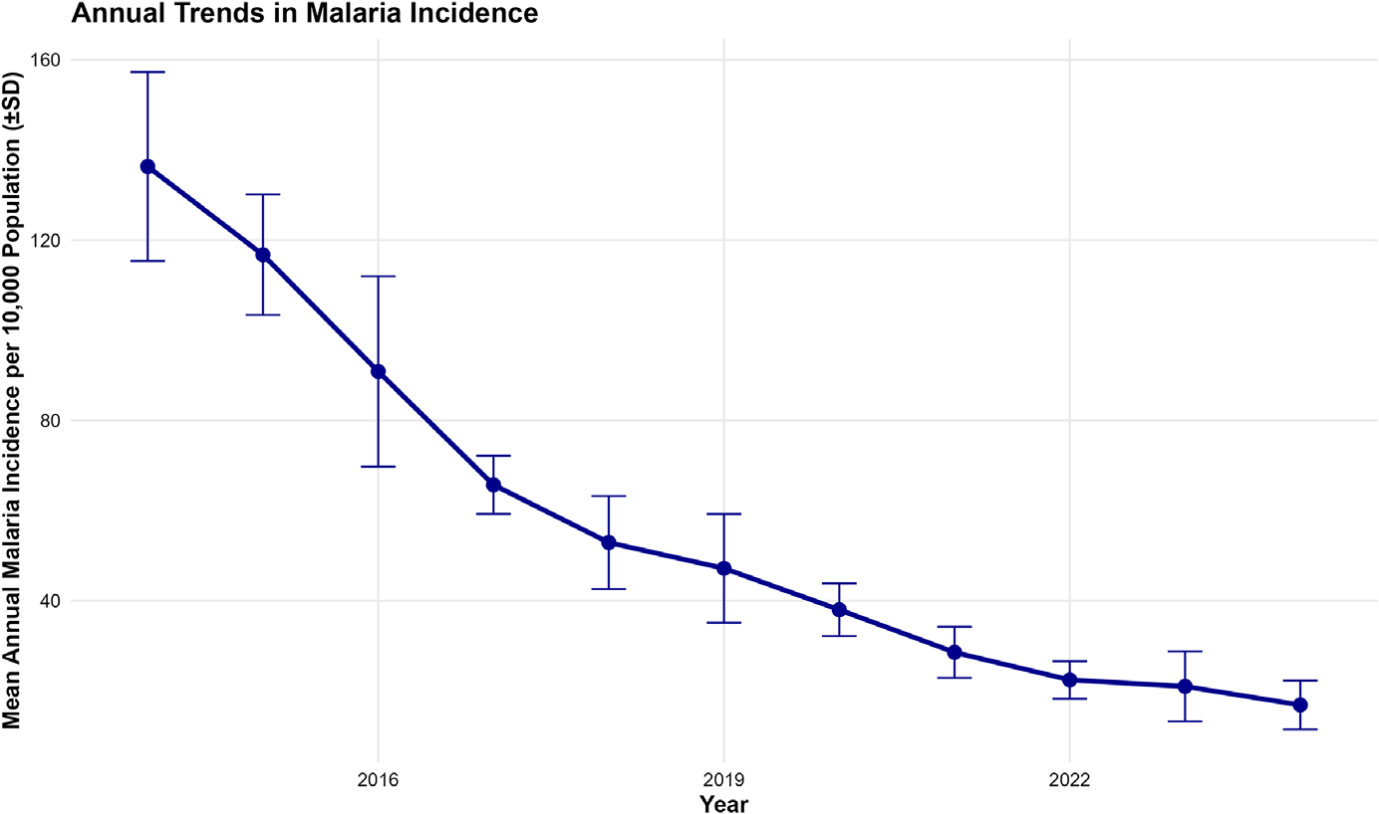
Annual trends of malaria incidence in Dar es Salaam (2014–2024). The interannual decline is statistically significant (*p* < 0.001). Figure 3. long description.

The monthly malaria incidence in Dar es Salaam from January 2014 to October 2024 exhibited a general declining trend over the study period (Figure 2). The time series demonstrated substantial month-to-month variability with peak incidence exceeding 180 cases per 10000 population in 2014–2015, while values decreased to below 50 cases per 10000 population by 2024. Annual mean malaria incidence showed a consistent downward trend from 2014 to 2024, with the highest mean yearly incidence recorded in 2014 (139.8 ± 25.3 cases per 10000) and the lowest observed in 2023 (22.4 ± 11.2 cases per 10000) (Figure 3).

### Patterns of climate variables

**Figure 4.**
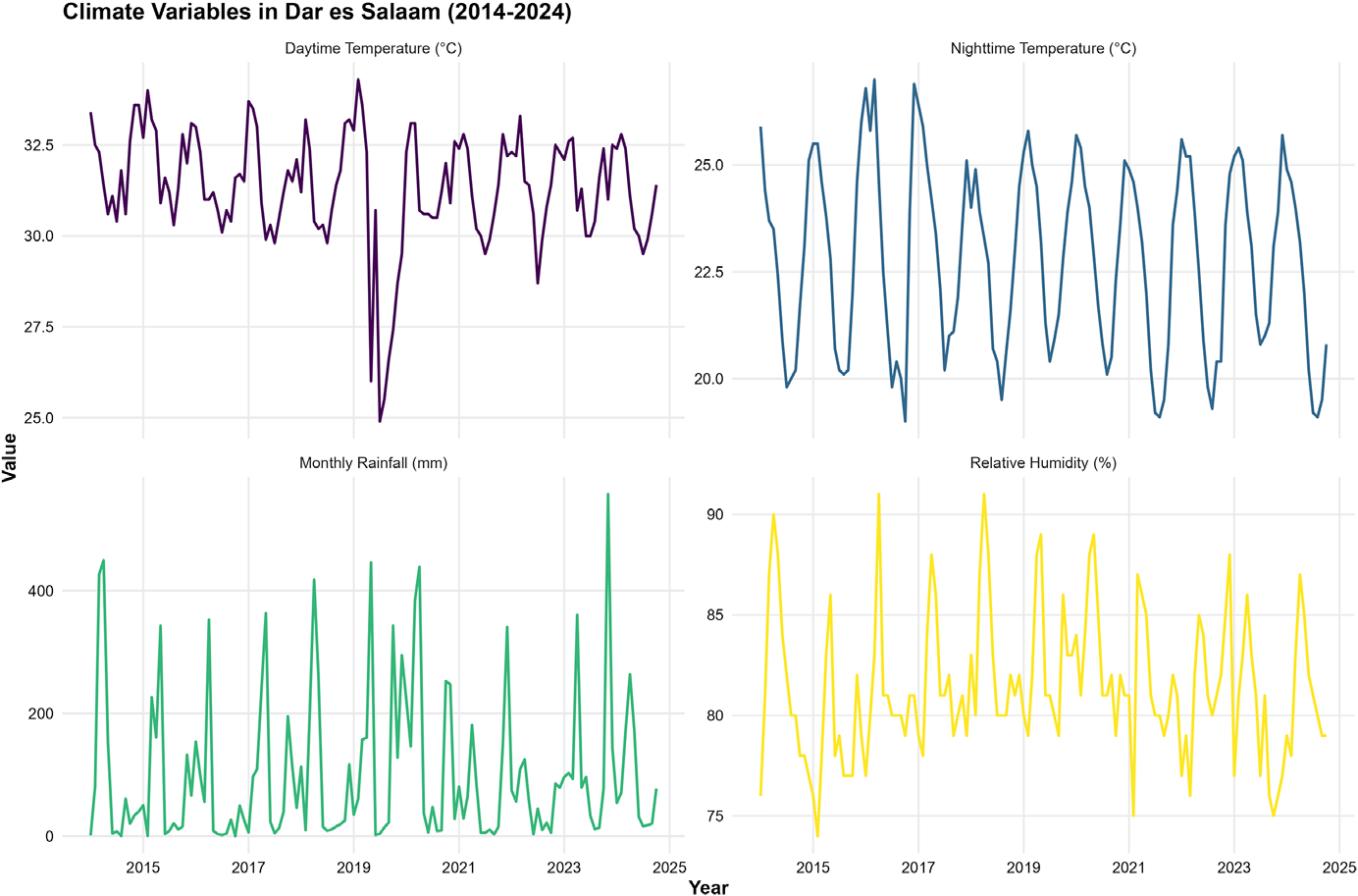
Time series plot for all considered climate variables. Figure 4. long description.

Daytime and night-time temperatures exhibited consistent seasonal patterns throughout the study period (Figure 4). Daytime temperatures ranged from approximately 26°C to 34°C, with peak values typically occurring during the short dry season (December–February). Night-time temperatures exhibited less variability, ranging from 19°C to 27°C, with similar seasonal patterns to daytime temperatures. Monthly rainfall exhibited high variability, with pronounced seasonal peaks during the long rainy season (March to May) and the short rainy season (October–December). Rainfall amounts ranged from near zero during dry periods to over 400 mm during peak rainy months. Relative humidity remained consistently high throughout the year, ranging from 75% to 91%, with slightly higher values during the rainy seasons.

### Seasonal trend of malaria incidence

**Figure 5.**
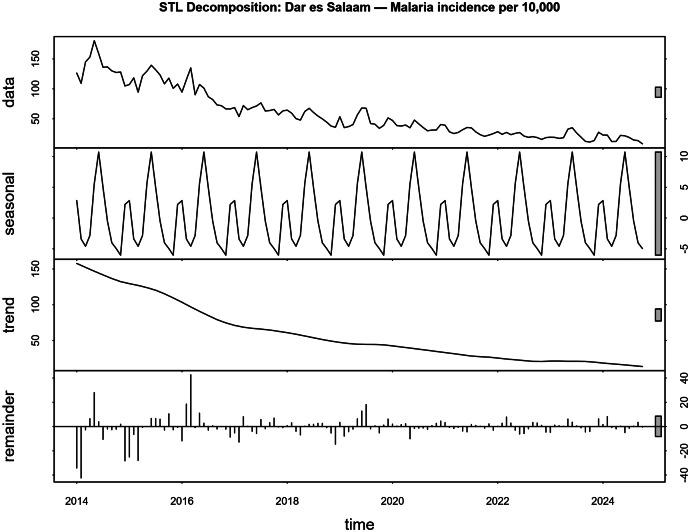
Seasonal-trend decomposition using locally weighted (STL) regression of monthly malaria incidence in Dar es Salaam (2014–2024). The panels from top to bottom display the original observed data, the estimated long-term trend, the extracted seasonal component, and the remainder (residuals). The *y*-axis represents the malaria incidence, and the *x*-axis represents the time in years. Figure 5. long description.

The STL decomposition of malaria incidence revealed three distinct components. The trend component showed an apparent long-term decline from 2014 to 2024, with the steepest decrease occurring between 2016 and 2018. The seasonal component exhibited a strong annual cycle with peak values during April–June and low values during August–September. The residual component demonstrated relatively low variability, indicating that the trend and seasonal components captured most of the temporal variation in malaria incidence (Figure 5).

**Figure 6.**
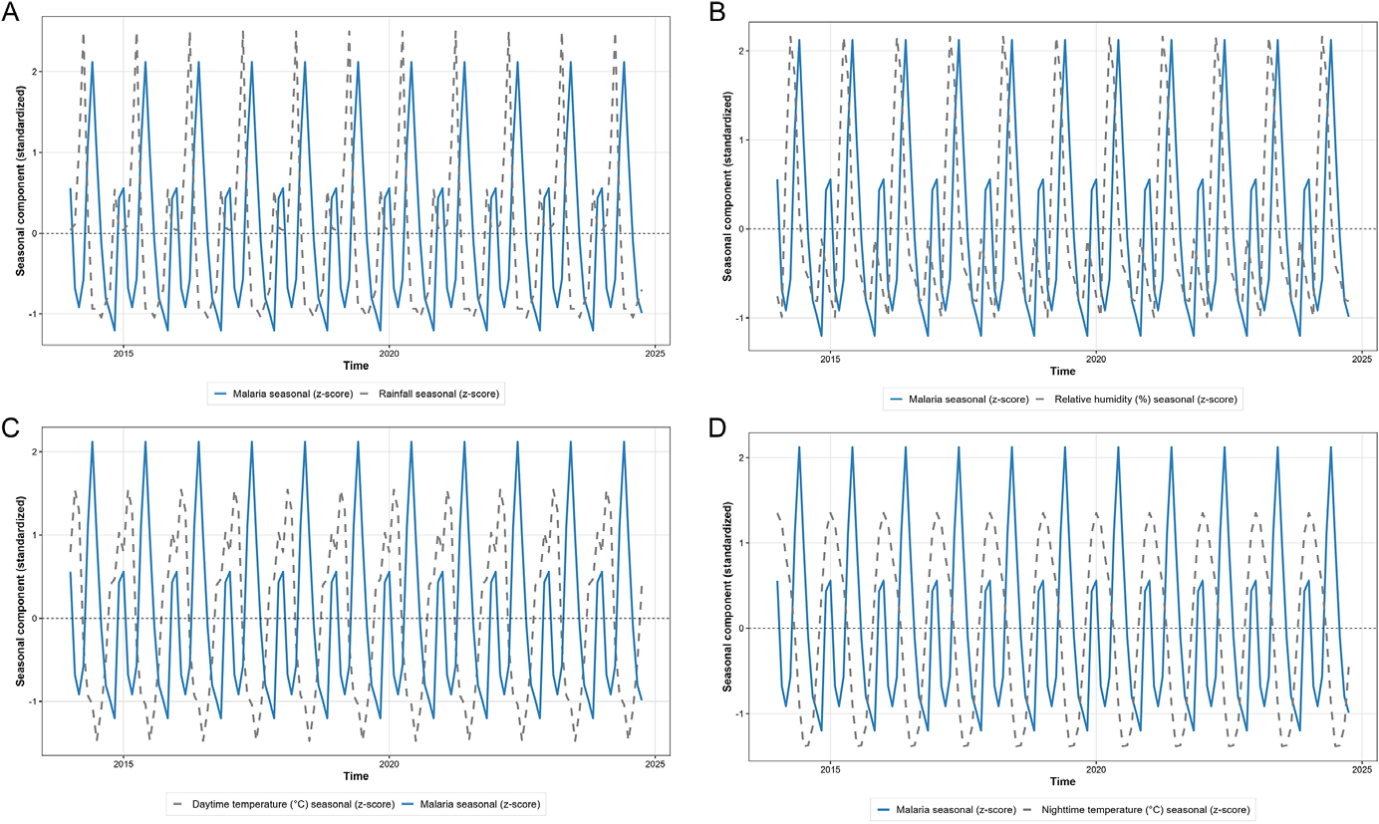
Standardized seasonal components of malaria incidence and climatic variables derived from STL decomposition. Panel A displays the seasonal trend of malaria incidence aligned with rainfall; Panel B shows with daytime temperature; Panel C shows with night-time temperature; and Panel D shows with relative humidity. The *y*-axis represents the standardized seasonal effect, demonstrating the 1- to 2-month lag between climatic peaks and malaria surges across the calendar year (*x*-axis). Figure 6. long description.

Seasonal-trend decomposition decomposition analysis comparing malaria incidence with climate variables revealed distinct seasonal relationships. The seasonal components revealed that malaria peaks lagged behind rainfall peaks by approximately 1–2 months, with rainfall seasonal peaks occurring in March and April, and malaria seasonal peaks occurring in April and May. Both daytime and night-time temperature seasonal patterns showed moderate association with malaria seasonality, with temperature peaks during the short dry season (December–February) preceding malaria peaks by 2–3 months. Relative humidity seasonal patterns revealed a similar lagged relationship to that of rainfall (Figure 6).

### Relationship between climatic variables and malaria incidence

The GAM model achieved an excellent fit, with an adjusted *R*^2^ of 0.975, explaining 97.9% of the deviance in malaria incidence (Table 1). The model included 127 observations spanning the entire period and utilized shrinkage cubic regression splines with restricted maximum likelihood (REML) estimation. The temporal trend component (year-month) was highly significant (edf = 7.633, F = 418.094, *p* < 0.001), confirming a substantial long-term decline in malaria incidence that is independent of climate effects. This component was included in the analysis structure to accurately capture trends.

**Table 1.** Associations between climatic factors and malaria incidence identified by generalized additive models Table 1. long description.

(A) Parametric term				
Term	Estimate	Std. error	*t* value	*p*-value
Intercept	56.8783	0.5517	103.1	<2e−16 ***

(B) Smooth terms (approximate significance)					
Smooth term	edf	Ref. df	F	*p*-value	Sig.
Daytime temperature	1.454	9	0.886	0.007	**
Nighttime temperature	0.000	9	0.000	0.628	
Monthly rainfall	0.000	9	0.000	0.607	
Relative humidity	2.093	9	0.928	0.012	*
Daytime temperature (lag 1 month)	3.428	9	2.501	<0.001	***
Daytime temperature (lag 2 months)	0.000	9	0.000	0.962	
Daytime temperature (lag 3 months)	0.000	9	0.000	0.367	
Night-time temperature (lag 1 month)	0.000	9	0.000	0.748	
Night-time temperature (lag 2 months)	1.288	9	0.385	0.024	*
Night-time temperature (lag 3 months)	0.396	9	0.064	0.144	
Rainfall (lag 1 month)	1.810	9	2.182	<0.001	***
Rainfall (lag 2 months)	1.316	9	2.517	<0.001	***
Rainfall (lag 3 months)	0.637	9	0.170	0.102	
Relative humidity (lag 1 month)	0.000	9	0.000	0.856	
Relative humidity (lag 2 months)	0.000	9	0.000	0.970	
Relative humidity (lag 3 months)	0.000	9	0.000	0.710	
Time (year–month)	7.633	11	418.094	<0.001	***

Family: Gaussian (identity). Smooths: shrinkage cubic regression splines (bs = ‘cs’), REML.

Model fit: Adjusted *R*^2^ = 0.975; Deviance explained = 97.9%; REML = 442.86; Scale = 38.651; *n* = 127.

Notes: edf, estimated degrees of freedom (non-linearity); Ref.df, reference df for *F*-test. Significance: ***p < 0.001; **p < 0.01; *p < 0.05.

**Figure 7.**
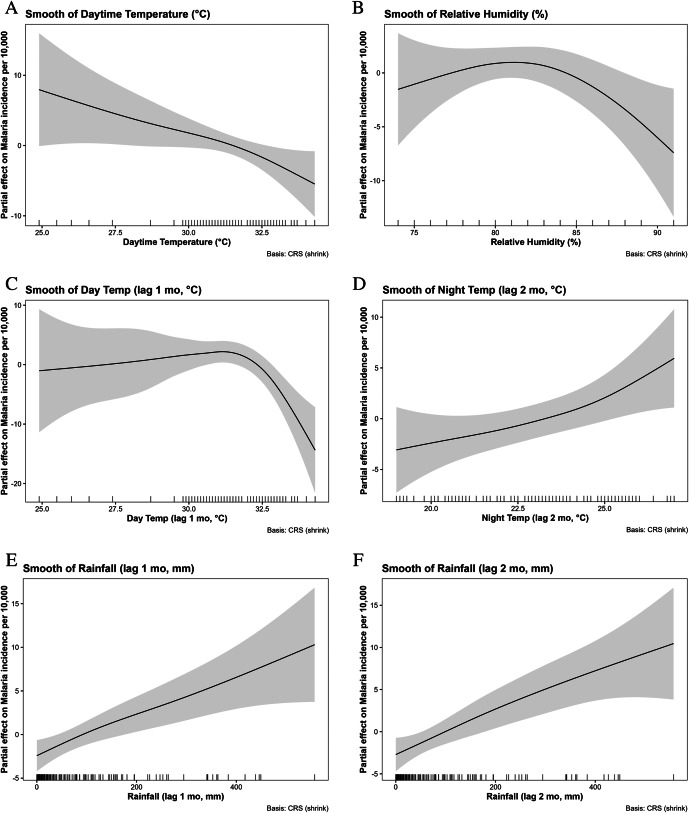
Non-linear associations between climatic factors and malaria incidence identified by generalized additive models: (a) Smooth of daytime temperature (°C); (b) Smooth of relative humidity (%); (c) Smooth of daytime temperature at 1-month lag (°C); (d) Smooth of night-time temperature at 2-month lag (°C); (e) Smooth of rainfall at 1-month lag (mm); and (f) Smooth of rainfall at 2-month lag (mm). Figure 7. long description.

Among the current month variables, daytime temperature exhibited a significant non-linear effect (edf = 1.454, *p* = 0.007), with malaria incidence declining steadily as temperatures rose from ~25°C to 33°C (Figure 7a). Relative humidity also had a significant non-linear effect (edf = 2.093, *p* = 0.012), showing a hump-shaped relationship in which incidence increased to around 80%–83% humidity before declining at higher values (Figure 7b). In contrast, the current month’s night-time temperature and rainfall showed no significant effects.

Lagged effects were more pronounced. Daytime temperature at a 1-month lag had the strongest association (edf = 3.428, *p* < 0.001), with a complex non-linear relationship: Incidence was relatively steady at cooler temperatures, rose to peak values around 31°C, and declined again thereafter (Figure 7c). Night-time temperature at a 2-month lag was also significant (edf = 1.288, *p* = 0.024), showing a broadly increasing relationship, with malaria incidence rising as night-time temperatures exceeded ~22°C (Figure 7d). Rainfall demonstrated robust positive associations at both 1- and 2-month lags (edf = 1.810, *p* < 0.001 and edf = 1.316, *p* < 0.001, respectively), with effects strengthening at higher monthly totals, particularly above 200 mm (Figures 7e–f).

Other lagged climate terms, including daytime temperature beyond 1-month lag, night-time temperature at 1 and 3 months, rainfall at 3 months, and lagged humidity, were not statistically significant and were effectively shrunk toward zero.

**Figure 8.**
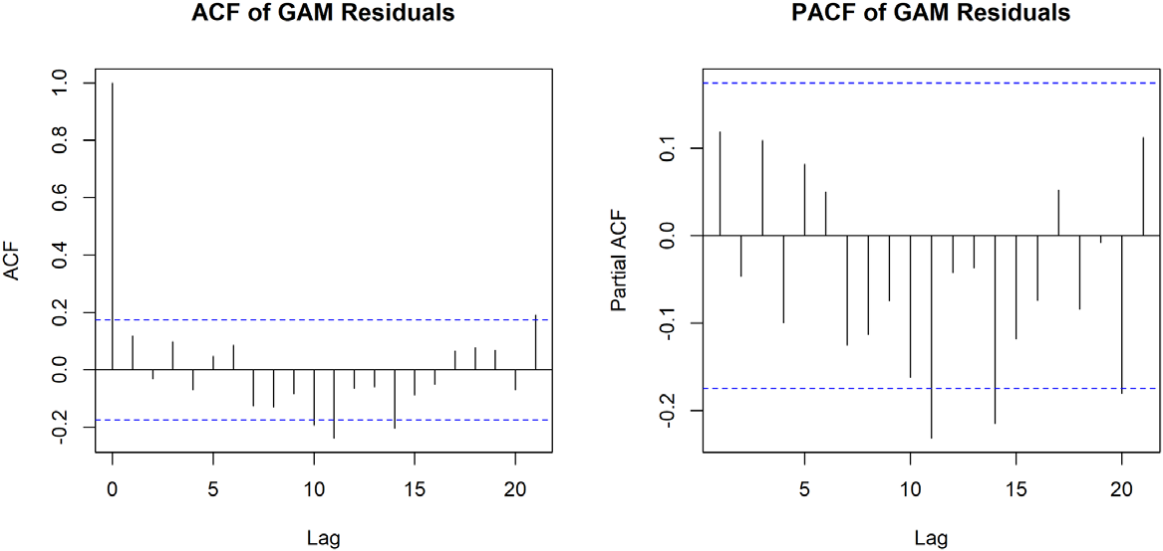
Autocorrelation function (ACF) and partial autocorrelation function (PACF) of residuals from the final generalized additive model (GAM) of monthly malaria incidence per 10000 population. Figure 8. long description.

Diagnostic checks of the model residuals confirmed the adequacy of the GAM fit. Evaluation of the autocorrelation function (ACF) and partial autocorrelation function (PACF) plots demonstrated that the residuals were independent, with values falling within the significance boundaries across all lags. This indicates that the inclusion of the highly flexible temporal spline successfully accounted for serial dependence and that no significant residual temporal autocorrelation remained in the model (Figure 8).

## Discussion

In this study, malaria incidence exhibited a general decline from January 2014 to October 2024. This downward trend aligns with findings from several studies conducted globally, which also reported reductions in malaria cases over time. For example, a study in Thailand revealed a non-linear decline in confirmed malaria cases, showing a significant decrease of 96.6% from 1061 cases in 2011 to only 36 cases in 2021 [17]. The study considered infections caused by *Plasmodium vivax and Plasmodium falciparum.* Similarly, in Korea, the confirmed infection rates of *P. vivax, P. malariae*, and *P. falciparum* showed a consistent downward trend throughout the study period [18]. These reductions were primarily attributed to differences in geographical conditions and effective control strategies. In contrast, approximately 96% of malaria cases in Tanzania are caused by *P. falciparum*, which remains the predominant species in the country.

A similar pattern was also observed in Northwest Ethiopia, where malaria prevalence declined by 68%, 60%, and 69% in 2017, 2018, and 2019, respectively, compared to 2014 [19]. The observed reductions were primarily attributed to intensified malaria control interventions, including the large-scale distribution of long-lasting insecticidal nets and the enhancement of surveillance systems. Despite this progress, malaria continues to pose a significant public health concern, particularly in regions where transmission remains persistent.

In contrast, some studies reported an increasing trend in malaria incidence over time. For instance, in Zanzibar, within Tanzania, malaria cases showed annual fluctuations between 2015 and 2020 but generally demonstrated an upward trend [20]. This resurgence may be attributed to the reduced effectiveness of interventions, increased importation of cases due to travel in this popular tourist destination, and the possible influence of climate variability and extreme weather events. Similarly, in the neighbouring country of the Democratic Republic of Congo, malaria prevalence increased steadily over 19 years (2001–2019), with an average prevalence rate of 13246 cases per 100000 individuals at risk, ranging from 1178383 to 1417483 cases [21]. This persistent increase was likely influenced by changing climatic conditions, including variations in rainfall and temperature, which create favourable environments for malaria transmission.

The STL decomposition revealed a strong annual seasonal cycle, with malaria incidence peaking between April and June and reaching its lowest levels between August and September. This pattern indicates higher malaria transmission during the long rainy season in Dar es Salaam. The period corresponds to the March–May (MAM) rains, with an extended peak into June, suggesting possible shifts in seasonal patterns that may influence malaria dynamics. The positive association between rainfall and malaria incidence is well established, as rainfall enhances mosquito breeding and survival conditions.

Similar seasonal trends have been documented elsewhere. In Australia and Papua New Guinea, malaria transmission increases during the rainy months when vector breeding sites expand [22]. In Pakistan, incidence peaks in March and declines in January and August [23], while in Burkina Faso, higher malaria prevalence between July and December has been observed, with September recording the highest incidence during 2013–2020 [24]. Comparable studies in Kenya and Uganda have also linked malaria surges to rainfall intensity and humidity, reinforcing the importance of climatic factors in shaping transmission patterns [25, 26].

The daytime temperature at a 1-month lag had the strongest association with malaria incidence among the variables assessed, exhibiting complex, non-linear relationships with peak values around 31°C, which declined thereafter. Moreover, nighttime temperature at a 2-month lag was also significantly associated with malaria incidence, showing a broadly increasing relationship, with incidence rising as nighttime temperatures exceeded ~22°C. Similarly, a study conducted in Asia and Africa found a positive linear correlation between the annual minimum temperature and the incidence of malaria in Africa [2]. Nonetheless, a strong negative correlation existed between malaria and temperature, particularly at 0- and 1-month lag [27]. Likewise, a study in China also exhibited a negative association between temperature and malaria incidence during the first 2 months [28].

The WHO emphasizes that the development and survival of *Anopheles* mosquitoes are strongly influenced by temperature. Optimal survival occurs between 20°C and 27°C, whereas mortality increases sharply beyond 28°C and becomes lethal above 38°C. Similarly, mosquito biting rates peak within the 20–30°C range but decline substantially below 15°C, reflecting the temperature dependence of the gonotrophic cycle. Notably, some species exhibit ‘gonotrophic discordance’, taking multiple blood meals before oviposition, which enhances transmission potential. Moreover, variations in anthropophilic, endophagic, and endophilic behaviours further influence human vector contact and malaria transmission dynamics [29].

Rainfall demonstrated robust positive associations at both 1- and 2-month lags, with effects strengthening at higher monthly totals, particularly above 200 mm. The relationship may also be influenced by changes in rainfall patterns, where unusual rainfall events result in the proliferation of mosquito breeding sites. Furthermore, mosquitoes reproduce rapidly, with their life cycle from egg to adult typically taking 7 to 14 days. However, depending on food availability and environmental conditions, this duration may prolong. Similarly, a study in Burkina Faso found a substantial positive correlation between malaria and precipitation, with malaria cases developing 1–3 months after precipitation episodes [30]. This dynamic highlights a critical distinction between Dar es Salaam and other East African urban centres. Unlike higher-altitude cities such as Nairobi, where temperature fluctuations are the primary limiting factor for transmission, coastal cities such as Dar es Salaam and Mombasa tend to exhibit transmission patterns more closely tied to rainfall variability and seasonality, which influence the availability of mosquito breeding habitats [31, 32]. However, the rapid urbanization of Dar es Salaam introduces specific micro-mechanisms such as increased impervious surfaces, inadequate drainage infrastructure, and artificial water accumulation that exacerbate these climatic effects. While our macro-level ecological design did not explicitly measure these urban micro-mechanisms, the robust 1- to 2-month lagged effect of heavy rainfall strongly suggests that urban water pooling creates highly productive, artificial breeding sites. Consequently, a core innovative finding of this study is the conclusive breakthrough that even amidst a 99% intervention-driven decline in a rapidly urbanizing megacity, fundamental non-linear climate sensitivities persist and sustain transmission.

In Ethiopia, a substantial positive association between malaria cases and precipitation with a 4-month lag has previously been reported. Furthermore, a strong contemporaneous relationship was found between rainfall and the overall number of malaria cases [33]. Conversely, studies across several Asia-Pacific countries, including Bangladesh, Bhutan, Indonesia, South Korea, Nepal, Thailand, and Vietnam, have shown that the incidence of malaria is negatively correlated with the annual precipitation [2]. This disparity may stem from differences in data resolution and context, as global climate datasets may not adequately capture localized climatic and ecological variations influencing malaria transmission.

In this study, relative humidity at all lag periods showed no significant association with malaria incidence. This finding suggests that even high humidity levels may not substantially influence malaria transmission when ambient temperatures remain below 18°C or exceed 35°C, as such conditions inhibit mosquito development and survival. Nonetheless, contrasting evidence from other regions suggests that humidity may play a significant role in malaria dynamics. Studies from Ethiopia and West Africa have reported positive associations between relative humidity and malaria incidence [7]. A 3-month lag in humidity significantly increased the overall number of malaria cases in Ethiopia [33].

### Limitations

A primary limitation of this study is the reliance on climate measurements from a single weather station (Julius Nyerere International Airport) to represent the entire 1350 km^2^ area of Dar es Salaam. The airport station was our best option because it provides the most complete, continuous, and World Meteorological Organization (WMO)-standardized meteorological dataset for the 10-year study period. Furthermore, because Dar es Salaam is situated on a relatively flat coastal plain, it does not experience the extreme elevation-driven microclimates seen in more mountainous East African cities. However, localized microclimatic differences – such as variations in humidity and rainfall between immediate coastal wards and inland suburban districts – inevitably exist. Consequently, relying on aggregated district-level data mapped to a single station may not fully capture these spatial heterogeneities and could underestimate small-area variations in climate-malaria relationships.

Furthermore, the lack of high-resolution spatio-temporal data for non-climatic factors prevented their direct inclusion as covariates. It is highly likely that the near 99% decline in malaria incidence observed during the study period was predominantly driven by the successful scale-up of vector control interventions. While the inclusion of a highly flexible temporal trend spline in our GAM successfully absorbed this long-term decline and statistically isolated the short-term climatic effects, this approach remains a proxy. Consequently, a major limitation of this study is the inability to explicitly model the interaction between intervention scale-ups and climate sensitivity. The lack of these explicit variables means we cannot fully uncouple the synergistic dynamics between aggressive vector control and changing environmental suitability, highlighting a critical area for future ecological studies.

Finally, the reliance on aggregated DHIS2 data introduces potential biases regarding diagnostic consistency and case reporting. While Tanzanian national guidelines mandate the use of standardized malaria rapid diagnostic tests (mRDTs) or microscopy across all health facilities, operational realities and diagnostic capacities inevitably vary between individual outpatient and inpatient institutions. Furthermore, disparities in health-seeking behaviours and potential under-reporting in lower-income suburban wards compared to affluent urban areas could introduce spatial bias, potentially affecting the accuracy of the calculated district-level incidence rates. Future studies incorporating localized socio-economic data and facility-level diagnostic audits are needed to resolve these discrepancies.

### Future research directions

This study relied solely on aggregated data from the DHIS2 system, which carries inherent limitations. Future research should consider hospital-based data and incorporate experimental study designs to generate more robust and detailed evidence. Moreover, subsequent studies should adopt a more holistic approach by integrating climate-related variables with other vital determinants such as hygiene and sanitation, vector control interventions, housing conditions, and host immunity. These factors may also play a significant role in shaping malaria incidence and should be examined in conjunction with climatic influences.

## Conclusion

This study highlights a massive, statistically significant decline in malaria incidence in Dar es Salaam over the past decade, dropping from a peak of over 130 cases per 10000 population in 2014 to fewer than 30 cases per 10000 by 2023. Despite this near 99% reduction, likely driven by the aggressive scale-up of vector control interventions, malaria transmission remains highly climate-sensitive and continues to exhibit strong seasonal surges. STL analysis revealed consistent annual peaks occurring between April and June, immediately following the March–May long rainy season.

Generalized additive models suggested that lagged rainfall is the most dominant climatic driver of this persistent transmission. Rainfall exhibited robust, positive associations at both 1- and 2-month lags (*p* < 0.001), with transmission risks accelerating when monthly rainfall exceeded 200 mm. Temperature also played a complex, non-linear role, with a 1-month lagged daytime temperature peaking in suitability at approximately 31°C. These findings underscore the pivotal role of rainfall-driven urban water pooling and highlight the urgent need for climate-informed early warning systems to target the remaining seasonal transmission pockets in rapidly urbanizing coastal cities.

## Data Availability

The dataset and R scripts supporting the findings of this study are publicly available via DOI:10.5281/zenodo.19425050. All relevant materials are accessible without restriction. For further information or inquiries, please contact the corresponding author.

## Long descriptions

### Figure 1 Long description

A map of the Dar es Salaam region divided into five administrative districts: Ilala, Kinondoni, Temeke, Ubungo, and Kigamboni. The districts are situated along the southwest coast of the Indian Ocean on a flat coastal plain.

Navigate back to Figure 1.

### Figure 2 Long description

A time series plot showing monthly malaria incidence per 10000 population. The data shows a substantial month-to-month variability with peak incidence exceeding 180 cases per 10000 in 2014–2015, declining to below 50 cases per 10000 by 2024.

Navigate back to Figure 2.

### Figure 3 Long description

A line plot showing the mean annual malaria incidence per 10000 population on the y-axis and the year on the x-axis, complete with standard deviation error bars. The graph illustrates a steady and significant downward trend, starting at approximately 140 cases per 10000 in 2014 and dropping to roughly 22 cases per 10000 by the year 2023.

Navigate back to Figure 3.

### Figure 4 Long description

A multi-panel time series plot tracking four climatic variables over a 10-year period. The top left panel shows daytime temperature fluctuating between 26°C and 34°C. The top right panel shows nighttime temperature fluctuating between 19°C and 27°C. The bottom left panel displays highly variable monthly rainfall, with cyclical peaks reaching over 400 mm. The bottom right panel shows relative humidity consistently ranging between 75% and 91%.

Navigate back to Figure 4.

### Figure 5 Long description

Four stacked line graphs decomposing the monthly malaria incidence from 2014 to 2024. The top panel shows the raw observed data. The second panel isolates the ‘seasonal’ component, revealing a highly regular annual cycle with distinct peaks and troughs. The third panel shows the ‘trend’ component, illustrating a smooth, continuous long-term decline over the decade. The bottom panel shows the ‘remainder’ or residuals, which cluster near zero.

Navigate back to Figure 5.

### Figure 6 Long description

A four-panel figure (A, B, C, D) mapping the standardized seasonal effect of malaria (solid line) alongside different climatic variables (dashed lines) over time. Panel A aligns malaria with rainfall; Panel B with daytime temperature; Panel C with nighttime temperature; and Panel D with relative humidity. The visualizations demonstrate a 1- to 2-month lag, where climatic peaks consistently precede the surges in malaria incidence across the calendar year.

Navigate back to Figure 6.

### Table 1 Long description

This table presents the results of the Generalized Additive Model (GAM) analysis. It indicates that the temporal trend (year-month), daytime temperature at a 1-month lag, and rainfall at one- and 2-month lags were highly statistically significant (p < 0.001) in explaining malaria incidence variability.

Navigate back to Table 1.

### Figure 7 Long description

Six panels (A through F) showing the partial effects of climatic factors on malaria incidence, with shaded 95% confidence intervals. Panel A shows a steady decline in incidence as current-month daytime temperature rises. Panel B shows a hump-shaped relationship for relative humidity. Panel C indicates incidence remains steady then sharply drops after daytime temperature (1-month lag) exceeds 31°C. Panel D shows incidence rising as nighttime temperature (2-month lag) increases. Panels E and F demonstrate robust, positive associations between rainfall and malaria incidence at 1-month and 2-month lags, respectively.

Navigate back to Figure 7.

### Figure 8 Long description

Two side-by-side bar plots displaying the ACF (left) and PACF (right) of the GAM residuals across 20 lags. Dashed blue lines indicate the significance boundaries. The vertical bars representing the residual values across the lags predominantly fall within these boundaries, indicating that no significant residual temporal autocorrelation remains in the model.

Navigate back to Figure 8.
